# Precise Regulation of the Basal PKCγ Activity by DGKγ Is Crucial for Motor Coordination

**DOI:** 10.3390/ijms21217866

**Published:** 2020-10-23

**Authors:** Ryosuke Tsumagari, Kenta Maruo, Sho Kakizawa, Shuji Ueda, Minoru Yamanoue, Hiromitsu Saito, Noboru Suzuki, Yasuhito Shirai

**Affiliations:** 1 Department of Applied Chemistry in Bioscience, Graduate School of Agricultural Sciences, Kobe University, Kobe 657-8501, Japan; gyama_rin5334@yahoo.co.jp (R.T.); 205a421a@stu.kobe-u.ac.jp (K.M.); uedas@people.kobe-u.ac.jp (S.U.); yamanoue@kobe-u.ac.jp (M.Y.); 2 Department of Biological Chemistry, Graduate School of Pharmaceutical Sciences, Kyoto University, Kyoto 606-8501, Japan; kakizawa.sho.4u@kyoto-u.ac.jp; 3 Department of Animal Functional Genomics of Advanced Science Research Promotion Center, Mie University Organization for the Promotion of Regional Innovation, Tsu 514-8507, Japan; hisaito@doc.medic.mie-u.ac.jp (H.S.); nsuzuki@doc.medic.mie-u.ac.jp (N.S.)

**Keywords:** DGKγ, PKCγ, LTD, motor coordination, TRPC3

## Abstract

Diacylglycerol kinase γ (DGKγ) is a lipid kinase to convert diacylglycerol (DG) to phosphatidic acid (PA) and indirectly regulates protein kinase C γ (PKCγ) activity. We previously reported that the basal PKCγ upregulation impairs cerebellar long-term depression (LTD) in the conventional DGKγ knockout (KO) mice. However, the precise mechanism in impaired cerebellar LTD by upregulated PKCγ has not been clearly understood. Therefore, we first produced Purkinje cell-specific DGKγ KO (tm1d) mice to investigate the specific function of DGKγ in Purkinje cells and confirmed that tm1d mice showed cerebellar motor dysfunction in the rotarod and beam tests, and the basal PKCγ upregulation but not PKCα in the cerebellum of tm1d mice. Then, the LTD-induced chemical stimulation, K-glu (50 mM KCl + 100 µM, did not induce phosphorylation of PKCα and dissociation of GluR2 and glutamate receptor interacting protein (GRIP) in the acute cerebellar slices of tm1d mice. Furthermore, treatment with the PKCγ inhibitor, scutellarin, rescued cerebellar LTD, with the phosphorylation of PKCα and the dissociation of GluR2 and GRIP. In addition, nonselective transient receptor potential cation channel type 3 (TRPC3) was negatively regulated by upregulated PKCγ. These results demonstrated that DGKγ contributes to cerebellar LTD by regulation of the basal PKCγ activity.

## 1. Introduction

Long-term depression (LTD) is one of the synaptic plasticity at synapses between parallel fibers (PFs) and Purkinje cells and is important for cerebellar motor coordination. LTD is induced by co-stimulation of PFs and climbing fiber (CF), which trigger the activation of metabotropic glutamate receptor 1 (mGluR1) signaling and the depolarization, leading to protein kinase C (PKC) activation [1,2,3]. PKC promotes the phosphorylation of Ser880 in the GluR2 subunit of α-amino-3-hydroxy-5-methylisoxazole-4-propionic acid (AMPA) receptor, causing clathrin-mediated endocytosis [4,5]. Among PKC subtypes, PKCα is required for the phosphorylation of Ser880 in GluR2, leading to LTD induction [6], and the spatial regulation of PKCα activity contributes to cerebellar LTD [7]. Although PKCγ is a major PKC isoform in Purkinje cells and PKCγ deficiency causes motor dyscoordination, cerebellar LTD was not impaired in PKCγ knockout (KO) mice [8,9]. On the other hand, constitutive active PKCγ mutant causes both the impairments of motor coordination and LTD [10]. These reports indicate that the precise regulation of PKC activity is responsible for cerebellar LTD and motor coordination.

Diacylglycerol kinase (DGK) is a lipid kinase terminating diacylglycerol (DG) signaling by converting DG to phosphatidic acid (PA) [11]. DG is an important lipid messenger that activates several enzymes including protein kinase C (PKC) [12]. PA also regulates various enzymes including mammalian target of rapamycin (mTOR) [13]. Therefore, DGK is thought to have the important physiological roles related to DG signaling. Among DGK subtypes, the γ isoform of DGK (DGKγ) is abundantly expressed in cerebellar Purkinje cells [14], and we recently reported that the conventional DGKγ KO mice showed impaired cerebellar LTD and motor dysfunction [15]. We also found abnormal upregulation of the basal PKCγ activity in DGKγ KO mice and that the PKCγ inhibitor normalized the impairment of LTD in DGKγ KO mice [15]. These results suggested that the functional correlation between DGKγ and PKCγ is responsible for LTD induction. However, it remains unknown how the upregulated PKCγ impairs cerebellar LTD in DGKγ KO mice.

In addition, although DGKγ also is abundantly expressed in other region of the brain including hippocampal pyramidal cells [14], the DGKγ KO mice used in the previous study lost the enzyme in the whole body. Therefore, in the present study, we newly produced Purkinje cell-specific DGKγ KO (tm1d) mice and investigated the specific function of DGKγ in Purkinje cells and the detailed mechanism of PKCγ in impaired LTD. We confirmed that Purkinje cell-specific DGKγ KO (tm1d) mice showed impairment of motor coordination similarly to the conventional DGKγ KO mice and found that the upregulated basal PKCγ activity negatively regulated PKCα inactivation during cerebellar LTD by the phosphorylation of a nonselective transient receptor potential cation channel type 3 (TRPC3). Our research demonstrates the important novel function of DGKγ and PKCγ in the cerebellar LTD and motor coordination.

## 2. Results

### 2.1. Motor Dyscoordination in Purkinje Cell-Specific DGKγ KO (tm1d) Mice

**Figure 1:**
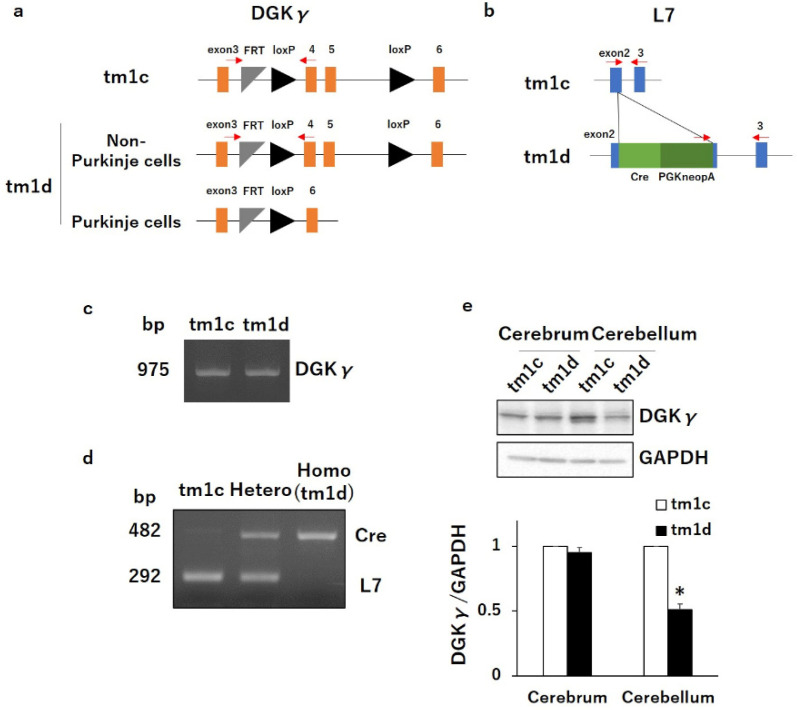
Generation of Purkinje cell-specific Diacylglycerol kinase γ (DGKγ) knockout (KO) (tm1d) mice and PCR genotyping: tm1c and tm1d alleles encodes DGKγ (**a**) and Cre recombinase knock-in L7 (**b**). Cre, Cre recombinase; FRT, flippase recognition target; loxP, Cre recombinase recognition sequence; PGKneopA, PGK-neomycin cassette. PCR genotyping of DGKγgene (**c**) and Cre recombinase (**d**) was determined using tail-derived genome: bands at 975, 482, and 292 bp were expected for the DGKγ, Cre, and L7 alleles, respectively. Red arrows show the primer sites for PCR genotyping. (**e**) Cerebral and cerebellar lysates from tm1c and tm1d mice were subjected to Western blotting and probed with an anti-DGKγ antibody. Quantification of the expression levels of DGKγ was performed by ImageJ. The expression level of DGKγ was normalized to the expression level of the loading control (Glyceraldehyde 3-phosphate dehydrogenase: GAPDH). (n = 3); * *p* <  0.05, followed by Student’s t-test. Data are expressed as mean ± SEM.

**Figure 2:**
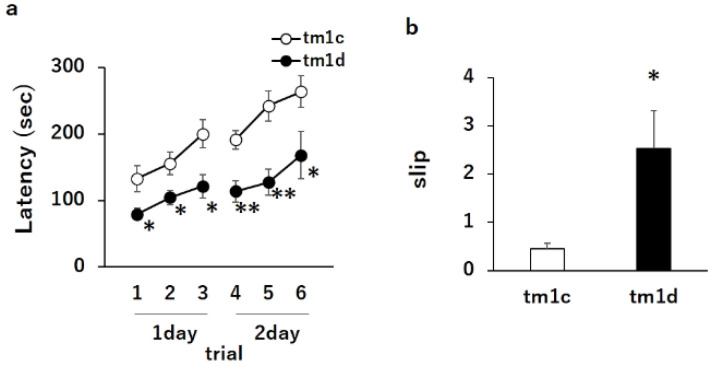
Motor dyscoordination in tm1d mice: (**a**) motor coordination of tm1c and tm1d mice at 16 weeks old was assessed by the accelerating rotarod test. The test was performed three times daily for 2 day (tm1c: n =  6; m1d: n =  5); * *p* < 0.05, ** *p* < 0.01, followed by Student’s t-test. (**b**) Motor coordination of tm1c and tm1d mice was assessed by the number of hind paw slips in the beam test. The test was performed five times daily for 2 day (tm1c: n = 6; tm1d: n = 5); * *p* < 0.05, followed by Student’s t-test. Data are expressed as mean ± SEM.

To investigate the specific function of DGKγ in Purkinje cells, we produced Purkinje cell-specific DGKγ KO (tm1d) mice by mating DGKγ floxed (tm1c) mice and L7/Pcp2-specific Cre recombinase transgenic mice using the Cre-loxP recombination system (Figure 1a,b). The genotypes of DGKγ gene and Cre gene were confirmed by PCR (Figure 1c,d). We examined the DGKγ expression levels in the brain of tm1d mice using western blotting and found that the DGKγ expression was significantly decreased at 16 weeks old (about 50%) in the cerebellum but not in the cerebrum (Figure 1e). The reason why the expression did not disappear completely was due to DGKγ expression in the granule cells [15]. Thus, we tested the motor coordination of tm1d mice by the rotarod and beam tests. In the rotarod test, tm1c and tm1d mice showed steady improvements over trials, but the latency for tm1d mice to fall from the rod was significantly shorter than that of tm1c mice (Figure 2a: tm1c 1, 133 ± 20.0; tm1d 1, 79 ± 8.8; tm1c 2, 155 ± 17.0; tm1d 2, 105 ± 10.9; tm1c 3, 200 ± 21.5; tm1d 3, 122 ± 17.6; tm1c 4, 191 ± 14.2; tm1d 4, 114 ± 15.9; tm1c 5, 242 ± 22.5; tm1d 5, 127 ± 19.4; tm1c 6, 264 ± 23.8; and tm1d 6, 168 ± 35.4). In the beam test, the tm1d mice showed more frequent slips than tm1c mice (Figure 2b: tm1c, 0.45 ± 0.12; tm1d, 2.53 ± 0.79). These results clearly indicated that the tm1d mice showed cerebellar motor dyscoordination.

### 2.2. PKCα Inactivation during LTD by Upregulated PKCγ Mediated Negative Regulation of TRPC3

**Figure 3:**
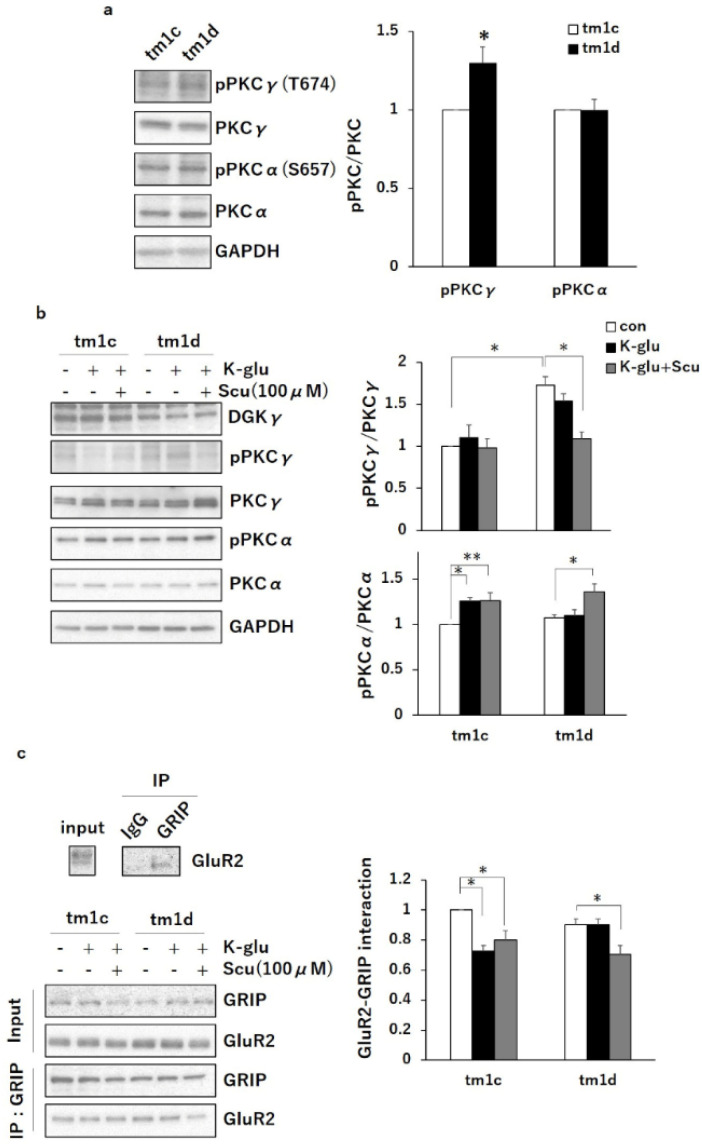
Protein kinase C α (PKCα) inactivation during long-term depression (LTD)-induced stimulation by K-glu in tm1d mice: (**a**) cerebellar lysates from tm1c and tm1d mice were subjected to Western blotting and probed with anti-PKCγ, anti-PKCα, anti-phospho-PKCγ, anti-phospho-PKCα, and anti-GAPDH antibodies. Quantification of the autophosphorylation of PKCγ and PKCα was performed by ImageJ. The phosphorylation levels of PKCγ and PKCα were normalized to the PKCγ and PKCα expression levels. The ratio of phosphorylation of PKCγ and PKCα to the expression levels of PKCγ and PKCα to tm1c was plotted (PKCγ: n = 3; PKCα: n = 3); * *p* < 0.05, followed by Student’s t-test. (**b**) Acute cerebellar slices from tm1c and tm1d mice were incubated with or without Scu (100 µm) for 1 h and subsequently were treated with K-glu (50 mM KCl + 100 µM L-glutamate) for 5 min. Lysates from the slices were subjected to Western blotting and probed with anti-DGKγ, anti-phospho-PKCγ, anti-PKCγ, anti-phospho-PKCα, anti-PKCα, and anti-GAPDH antibodies. Quantification of the autophosphorylation of PKCγ and PKCα was performed by ImageJ. The phosphorylation levels of PKCγ and PKCα were normalized to the PKCγ and PKCα expression levels. The ratio of phosphorylation of PKCγ and PKCα to the control (con) in tm1c was plotted (n = 6); * *p* < 0.05, ** *p* < 0.01, followed by Tukey’s multiple comparisons test. (**c**) The lysates after K-glu treatment were immunoprecipitated using anti-glutamate receptor interacting protein (GRIP) antibody. The immunoprecipitates were subjected to western blotting and probed with anti-GRIP and anti-GluR2 antibodies. IgG (anti-transient receptor potential cation channel type 3 (TRPC3) antibody) was used as a control. Quantification of co-immunoprecipitated GluR2 was performed by ImageJ and was normalized to the input level of GluR2. The ratio of the co-immunoprecipitated GluR2 to the control (con) in tm1c was plotted (n = 6); * *p* < 0.05, followed by Tukey’s multiple comparisons test. Data are expressed as mean ± SEM. Con means control.

We previously reported that PKCγ activity is regulated by DGKγ and that the upregulation of basal PKCγ causes motor dyscoordination [15,16]. To investigate the molecular mechanism of motor dyscoordination in tm1d mice, we examined the autophosphorylation level of PKCγ as a hallmark of PKC activity in the cerebellum of tm1d mice. Western blotting revealed that the phosphorylation level of PKCγ was higher in the cerebellum of tm1d mice than in that of tm1c mice (Figure 3a: tm1c, 1.00; tm1d, 1.30 ± 0.10). Also, we checked the basal phosphorylation level of PKCα based on previous studies demonstrating that PKCα plays a critical role in motor coordination [6,17]. However, the basal phosphorylation level of PKCα in tm1d mice was equivalent to that in tm1c mice (Figure 3a: tm1c, 1.00; tm1d, 1.00 ± 0.070). These results suggested that the upregulation of basal PKCγ activity in tm1d mice causes the impairment of motor coordination, similarly seen in the conventional DGKγ KO mice [15].

LTD stimulation induced the activation of PKCα, which is important for the expression of cerebellar LTD [6]. Therefore, we investigated whether PKCα was activated during LTD in tm1d mice, although the basal PKCα activity was not changed. We prepared cerebellar slices (300 μm) from tm1c and tm1d mice and chemically induced cerebellar LTD with K-glu (50 mM KCl + 100 µM Glu) treatment for 5 min. K-glu did not affect the phosphorylation level of PKCγ in tm1c and tm1d mice, indicating that PKCγ activity is not changed during LTD in both mice (Figure 3b: tm1c con, 1.00; tm1c K-glu, 1.10 ± 0.15; tm1d con, 1.73 ± 0.11; tm1d K-glu, 1.54 ± 0.092). On the other hand, K-glu induced the PKCα phosphorylation in the cerebellar slices from tm1c mice but not in that from tm1d mice (Figure 3b: tm1c con, 1.00; tm1c K-glu, 1.25 ± 0.037; tm1d con, 1.07 ± 0.038; tm1d K-glu, 1.10 ± 0.068). Furthermore, we examined the interaction between glutamate receptor interacting protein (GRIP) and GluR2 in the cerebellum because the phosphorylation of GluR2 by PKCα induces the dissociation of GluR2 from GRIP, which is required for LTD induction [18]. The co-immunoprecipitates with the anti-GRIP antibody from cerebellar slices showed the dissociation of GluR2 from GRIP in tm1c mice during LTD but not in tm1d mice (Figure 3c: tm1c con, 1.00; tm1c K-glu, 0.72 ± 0.041; tm1d con, 0.90 ± 0.039; tm1d K-glu, 0.90 ± 0.039). These results indicated that PKCα in tm1d mice was not activated during LTD. Then, to investigate the involvement of abnormal basal PKCγ activation in the impaired PKCα phosphorylation during LTD in tm1d mice, we examined the effect of a PKCγ inhibitor, scutellarin. Scutellarin normalized the upregulation of basal PKCγ activity in tm1d mice to control (tm1c) level and did not affect the autophosphorylation level of PKCγ in tm1c mice (Figure 3b: tm1c con, 1.00; tm1c K-glu+Scu, 0.98 ± 0.11; tm1d con, 1.72 ± 0.11; tm1d K-glu+Scu, 1.09 ± 0.079). Furthermore, the scutellarin treatment normalized the K-glu-induced PKCα phosphorylation and the dissociation of GluR2 from GRIP during LTD in tm1d mice (Figure 3b: tm1d K-glu, 1.10 ± 0.068; tm1d K-glu+Scu, 1.36 ± 0.089, c: tm1d K-glu, 0.90 ± 0.039; tm1d K-glu+Scu, 0.71 ± 0.058). These results indicated that the upregulated PKCγ activity in the basal state inhibits PKCα activation during LTD in tm1d mice.

**Figure 4:**
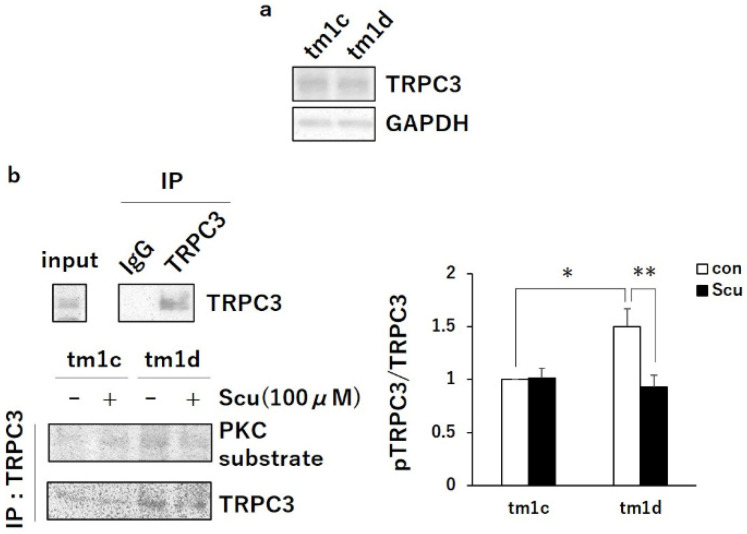
TRPC3 upregulation in tm1d mice: (**a**) cerebellar lysates from tm1c and tm1d mice were subjected to Western blotting and probed with anti-TRPC3 and anti-GAPDH antibodies. (**b**) Acute cerebellar slices from tm1c and tm1d mice were incubated with or without Scu (100 µM) for 1 h and subsequently were homogenized. Then, the lysates were immunoprecipitated using anti-TRPC3 antibody. The immunoprecipitates were subjected to western blotting and probed with anti-PKC substrate and anti-TRPC3 antibodies. IgG (anti-GRIP antibody) was used as a control. Quantification of the phosphorylation level of TRPC3 was performed by ImageJ and was normalized to the total level of TRPC3. The ratio of the phosphorylation level of TRPC3 to the control (con) in tm1c was plotted (n = 7); * *p* < 0.05, ** *p* < 0.01, followed by Tukey’s multiple comparisons test. Data are expressed as mean ± SEM. Con means control.

Next, we focused on a calcium channel, TRPC3, based on the following reports. PKCα activation during LTD requires intracellular Ca^2+^ increase [19], and TRPC3, which is abundantly expressed in Purkinje cells, is important for motor coordination and LTD [20,21]. Furthermore, PKCγ negatively regulates TRPC3 activity and the extracellular Ca^2+^ influx by the phosphorylation in Ser712 [22,23]. Therefore, we compared the phosphorylation level of TRPC3 in the cerebellum of tm1d and tm1c mice. The immunoprecipitates with the anti-TRPC3 antibody from cerebellar slices revealed that the phosphorylation level of TRPC3 increased in tm1d mice compared to in tm1c mice, although there was no differences in the expression levels of TRPC3 in the cerebellum of tm1c and tm1d mice (Figure 4a,b: tm1c -Scu, 1.00; tm1d -Scu, 1.50 ± 0.17). Importantly, scutellarin normalized the phosphorylation level of TRPC3 in tm1d mice to the level in tm1c mice (Figure 4b: tm1d -Scu, 1.50 ± 0.17; tm1d +Scu, 0.93 ± 0.11). This result indicated that the inactivation of TRPC3 by the upregulated PKCγ activity inhibited PKCα activation during LTD, leading to cerebellar motor dysfunction in tm1d mice.

## 3. Discussion

In this study, we confirmed the importance of DGKγ in motor coordination and the abnormal PKCγ upregulation at the basal state using Purkinje cell-specific DGKγ KO (tm1d) mice. In addition, we found that the impaired PKCα activation during K-glu impaired LTD in the cerebellar slices from tm1d mice. Similar impairment of PKCα activation during LTD was also detected in the cerebellar slices from the conventional DGKγ KO mice, which showed impaired LTD and motor dyscoordination (data not shown). Previously, we showed that LTD in the conventional DGKγ KO mice was rescued by a PKCγ inhibitor. These results strongly suggested that the upregulated PKCγ in both the conventional and Purkinje cell-specific DGKγ KO mice somehow inhibited PKCγ during LTD, resulting in motor dyscoordination.

**Figure 5:**
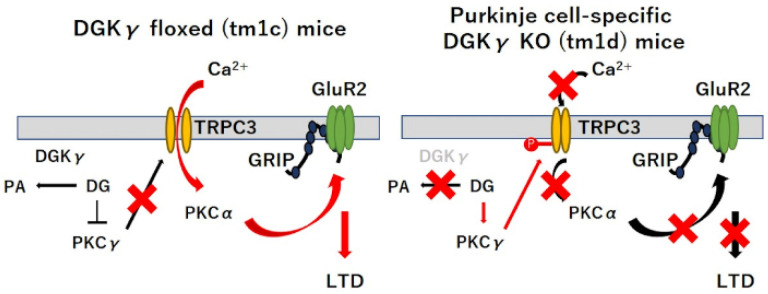
The proposed model of the function of DGKγ in the regulation of PKCγ.

PKCα activation to induce cerebellar LTD requires the transient increase in the internal Ca^2+^ level by coactivation of mGluR1 signaling through PFs and the depolarization through CF, which induce Ca^2+^ release from the intracellular stores in endoplasmic reticulum (ER) and Ca^2+^ influx via voltage-dependent calcium channels (VDCCs), respectively [19,24]. TRPC3 also is activated by mGluR1 signaling, inducing a slow excitatory postsynaptic current (EPSC) and a local dendritic Ca^2+^ signal [25,26], and we found that TRPC3 is negatively regulated by upregulated PKCγ in tm1d mice in this study, indicating the importance of TRPC3-mediated Ca^2+^ signaling in Purkinje cells as well as other reports [20,21,25,27]. In addition, the disorder of TRPC3 causes cerebellar motor dyscoordination and impaired LTD, although Ca^2+^ influx through TRPC3 is 2 to 4 less than the Ca^2+^ release form ER [28], and recent study reported that TRPC3 activity affected vascular endothelial growth factor (VEGF)-induced PKCα activation in human primary endothelial cells [29]. These results supported our conclusion that the negative regulation of TRPC3 by upregulated PKCγ in the basal state inhibits PKCα activation during LTD, resulting in motor dyscoordination (Figure 5). In other words, DGKγ was responsible for cerebellar LTD by the regulation of the basal PKCγ activity. On the other hand, PKCγ also regulates DGKγ activity. PKCγ directly interacts with DGKγ and phosphorylates DGKγ, leading to DGKγ activation. Activated DGKγ metabolizes DG and subsequently inactivates PKCγ [16]. These results suggested that DGKγ and PKCγ mutually regulate each other’s activity and that such functional correlation is important for cerebellar coordination.

In addition to Purkinje cells, DGKγ also is expressed in hippocampal pyramidal cells [14,15], suggesting that DGKγ is important for synaptic plasticity in hippocampal neuron. Many research reported DGK subtypes are involved in several forms of hippocampal synaptic plasticity [30]. In addition, PKCγ was also abundantly expressed in hippocampal pyramidal cells and PKCγ deficiency causes deficits in LTP and in spatial and contextual learning [31,32]. Therefore, DGKγ may also be involved in hippocampal synaptic plasticity by regulation PKCγ activity.

Not only DGKγ and PKCγ but also other DGKs and PKCs are involved in synaptic plasticity [33]. To date, 10 mammalian subtypes of DGK have been identified [34,35,36], and at least 8 subtypes are expressed in central nervous system (CNS) [37,38]. For example, DGKζ physically interacts with PKCα, reducing PKCα activity in the basal state, which is responsible for cerebellar LTD [39]. Additionally, DGKε also regulates cerebellar motor coordination by DG metabolization with DG lipase a (DGLα) [40]. DGKβ was characteristically localized at the plasma membrane, which is the hallmark of DGK activation [41]. DGKβ KO mice showed downregulated postsynaptic long-term potentiation (LTP) at Schaffer-collateral (SC)-CA1 synapses, resulting in impaired cognitive function and hyperactivity [42,43]. DGKι regulates mGluR-dependent LTD at SC-CA1 synapses [44], and DGKζ and DGKκ are responsible for both LTP and LTD at these synapses [45,46]. These results suggest that DGKs function as modulators in synaptic plasticity through regulating PKC activity.

In summary, we demonstrated that the precise regulation of the basal PKCγ activity by DGKγ is crucial for cerebellar LTD. In the basal state, DGKγ downregulated PKCγ activity by yielding PA from DG, which is the activator of PKCγ. LTD stimulation can induce PKCα activation by the extracellular Ca^2+^ influx through TRPC3, leading to internalization of GluR2 and cerebellar LTD. However, in the Purkinje cell-specific DGKγ KO (tm1d) mice, upregulated PKCγ by DGKγ deficiency inactivates TRPC3, leading to PKCα inactivation by the inhibition of Ca^2+^ influx during LTD stimulation. Our results provide a new understanding into the mechanism underlying cerebellar LTD.

## 4. Materials and Methods

### 4.1. Materials

Primers were purchased from Thermo Fisher scientific (Waltham, MA, USA). We used the following antibodies: rabbit anti-DGKγ [14], rabbit anti-PKC substrate (#2261) (Cell Signaling, Beverly, MA, USA), rabbit anti-phosphor-PKCγ T674 (bs-3730R) (Bioss, Woburn, MA, USA), rabbit anti-phosphor-PKCαS657 (ab180848) (Abcam, Cambridge, UK), rabbit anti-PKCγ (sc-211), mouse anti-PKCα (sc-8393), mouse anti-TRPC3 (sc-514670), mouse anti-GRIP antibody (sc-365937), mouse anti-GluR2 antibody (sc-517265), mouse anti-GAPDH (sc-47724) (Santa Cruz, Dallas, TX, USA), and peroxidase-conjugated AffiniPure goat anti-rabbit and mouse IgG. Scutellarin was purchased from Namiki Shoji (Tokyo, Japan).

### 4.2. Mice

DGKγ floxed (tm1c) mice were used as previously reported [15]. *L7/Pcp2*-specific Cre recombinase transgenic mice were kindly provided by Dr. Suzuki [47]. Purkinje cell-specific DGKγ (tm1d) mice were generated by mating tm1c mice with *L7/Pcp2*-specific Cre recombinase transgenic mice using the Cre-*loxP* recombination system. These mice were housed under a 12-h light, 12-h dark cycle with ad libitum food and water. All procedures using mice were performed according to the guidelines of the Institute Animal Care and Use Committee of Kobe University.

### 4.3. Genotyping

We used the tail-derived genome for PCR genotyping. Genotyping of DGKγ gene was determined by PCR using the following primers: 5′-CAGGTGTCTCTTGTCTGGGCT-3′ and 5′-TGGGTATAGGGTAGGAACTTGC-3′. Bands at 975 bp are expected from the DGKγ gene. Genotyping of the Cre recombinase gene was conducted by PCR using the following primers: 5′-ATATCCATGAGATTGTCCAT-3′, 5′-GAAGGCTTCTTCAACCTGCT-3′ and 5′-GACGTGCTACTTCCATTTGT-3′. Bands at 292-bp and 482-bp are expected from the L7 gene and Cre gene, respectively. PCR conditions were as follows: 25 μL volume, 1 cycle at 94 °C for 2 min; 40 cycles at 94 °C for 1 min, 62 °C (DGKγ) or 57 °C (Cre) for 30 s, and 72 °C for 1 min; and 1 cycle at 72 °C for 10 min.

### 4.4. Western Blotting

The cerebellum and cerebrum were homogenized in 500 μL ice-cold homogenate buffer (in mM: 20 Tris-HCl, 1 ethylene glycol tetraacetic acid (EGTA), 1 ethylenediaminetetraacetic acid (EDTA), 1 MgCl_2_, and 1 phenylmethylsulfonyl fluoride (PMSF), 20 ng/mL leupeptin, 1 × phosphatase inhibitor cocktail solution Ⅱ (Wako, Osaka, Japan), and 1% Triton X-100, pH 7.4) using Handy Sonic Sonicator (UR-20, Tomy Seiko Co., Ltd., Tokyo, Japan.). After centrifugation at 10,000 rpm for 10 min at 4 °C, the lysates were obtained.

Western blotting was performed as described previously [41]. Briefly, the samples (10 μg protein) were subjected to 10% SDS-PAGE, followed by blotting onto a poly-vinylidene difluoride membrane (Millipore, Darmstadt, Germany). Nonspecific binding sites were blocked by incubation with 5% skim milk in 0.01 M phosphate buffered saline (PBS) containing 0.03% TritonX-100 (PBS-T) for 1 h. The membrane was incubated with the appropriate antibody for 1 h at room temperature. After washing with PBS-T, the membrane was incubated with peroxidase-labeled anti-rabbit or mouse IgG for 30 min. After three rinses with PBS-T, the immunoreactivity bands were visualized using ImmunoStar (Wako, Osaka, Japan). The densities of the bands were analyzed by ImageJ. To detect phosphorylated protein, we used 2% bovine serum albumin (BSA) instead of skim milk for blocking and 0.01 M tris buffered saline (TBS) containing 0.03% Tween 20 (TBS-T) instead of PBS-T.

### 4.5. Rotarod Test

The rotarod apparatus (MK-630B single lane rotarod, Muromachi Kikai Co., LTD., Tokyo, Japan) consisted of a rod (30 mm in diameter, 90 mm wide) flanked by two large round plates (40 cm in diameter). The speed of rotation was increased from 4 to 40 rotation per minute (rpm) over 5 min and then remained at 40 rpm for an additional 300 s was maintained for 300 s. We recorded latency for the mice to fall from the rod. The test was performed 3 times daily for 2 days

### 4.6. Beam Test

Mice were trained to traverse elevated metallic beam (70 cm long, 10 mm in diameter, 60 cm high). They were placed at one end of the beam, and an enclosed escape box was placed at the other end. Each hind paw slip was recorded and counted. The test was performed 5 times daily for 2 days.

### 4.7. Acute Cerebellar Slice

Acute parasagittal cerebellar slices (300 µm thick) were prepared from the vermis [48,49,50]. The slices were incubated in artificial cerebrospinal fluid (ACSF) for 30 min at 37 °C and then incubated 1 h at room temperature in standard ACSF containing picrotoxin (100 µM). Scutellarin (100 µM) was added into the extracellular solution for 1 h. After that, to chemically induce LTD, the slices were treated with K-glu (50 mM KCl + 100 µM glutamate) for 5 min. Then, the slices were subsequently homogenized in 100 µL lysis buffer (50 mM Tris–HCl, 150 mM NaCl, 1 mM EDTA, 1 mM PMSF, 20 ng/mL leupeptin, 100 mM NaF, 2 mM Na_3_VO_4_, 20 mM Na_4_P_2_O_7_, 1% Triton X-100, and 10% glycerol, pH 7.5) using Handy Sonic Sonicator. After centrifugation at 10,000 rpm for 10 min at 4 °C, the lysates (10 µg protein) were subjected to western blotting.

For GRIP-GluR2 interaction, after the slices (300 µm thick) were homogenized with 100 µL lysis buffer and centrifugated, the lysates (500 µg protein) were diluted to 1 mL with homogenate buffer and 0.4 µg anti-GRIP antibody was added to the lysates. IgG (anti-TRPC3 antibody) was used as a control. Then, the lysates were rotated overnight at 4 °C followed by the addition of 10 µL Protein G sepharose and rotated for 2 h at 4 °C. After centrifugation at 10,000 rpm for 1 min at 4 °C for wash out with homogenate buffer, the immunoprecipitates were subjected to western blotting.

For TRPC3 phosphorylation, freshly prepared cerebellar slices (300 µm thick) were incubated in ACSF for 30 min at 37 °C and then incubated 1 h at room temperature in standard ACSF containing picrotoxin (100 µM) and scutellarin (100 µM). After the slices were then homogenized with 100 µL lysis buffer and centrifugated, the lysates (500 µg protein) were diluted to 1 mL with PBS-T and 0.4 µg anti-TRPC3 antibody was added to the lysates. IgG (anti-GRIP antibody) was used as a control.

Then, the lysates were rotated for 2 h at 4 °C followed by the addition of 10 µL Protein G sepharose and rotated for 1 h at 4 °C. After centrifugation at 10,000 rpm for 1 min at 4 °C for wash out with PBS-T, the immunoprecipitates were subjected to western blotting.

### 4.8. Experimental Design and Statistical Analysis

All animal data were analyzed for male mice. All data are shown as the means ± SEM, and Student’s t-tests and Tukey’s multiple comparison test were used as appropriate to test statistical significance. Data were analyzed using Excel (Microsoft, Seattle, WA, USA) and R version 3.5.1 (The R Foundation for Statistical Computing, Vienna, Austria). Differences were considered significant when *p* < 0.05.

## Abbreviations

AMPA	α-amino-3-hydroxy-5-methylisoxazole-4-propionic acid
CF	Climbing fiber
CNS	Central nervous system
DGK	Diacylglycerol kinase
DGL	Diacylglycerol lipase
EPSC	Excitatory postsynaptic current
GRIP	Glutamate receptor interacting protein
K-glu	KCl-L-glutamate
LTD	Long-term depression
LTP	Long-term potentiation
mGluR1	Metabotropic glutamate receptor 1
mTOR	Mammalian of target of rapamycin
PA	Phosphatidic acid
PFs	Parallel fibers
PKC	Protein kinase C
SC	Schaffer-collateral
TRPC3	Nonselective transient receptor potential cation channel type 3
VDCCs	Voltage dependent calcium channels
